# JCoast – A biologist-centric software tool for data mining and comparison of prokaryotic (meta)genomes

**DOI:** 10.1186/1471-2105-9-177

**Published:** 2008-04-01

**Authors:** Michael Richter, Thierry Lombardot, Ivaylo Kostadinov, Renzo Kottmann, Melissa Beth Duhaime, Jörg Peplies, Frank Oliver Glöckner

**Affiliations:** 1 Microbial Genomics Group, Max Planck Institute for Marine Microbiology, Celsiusstrasse 1, D-28359 Bremen, Germany; 2 Jacobs University Bremen gGmbH, D-28759 Bremen, Germany; 3 Ribocon GmbH D-28359 Bremen, Germany

## Abstract

**Background:**

Current sequencing technologies give access to sequence information for genomes and metagenomes at a tremendous speed. Subsequent data processing is mainly performed by automatic pipelines provided by the sequencing centers. Although, standardised workflows are desirable and useful in many respects, rational data mining, comparative genomics, and especially the interpretation of the sequence information in the biological context, demands for intuitive, flexible, and extendable solutions.

**Results:**

The JCoast software tool was primarily designed to analyse and compare (meta)genome sequences of prokaryotes. Based on a pre-computed GenDB database project, JCoast offers a flexible graphical user interface (GUI), as well as an application programming interface (API) that facilitates back-end data access. JCoast offers individual, cross genome-, and metagenome analysis, and assists the biologist in exploration of large and complex datasets.

**Conclusion:**

JCoast combines all functions required for the mining, annotation, and interpretation of (meta)genomic data. The lightweight software solution allows the user to easily take advantage of advanced back-end database structures by providing a programming and graphical user interface to answer biological questions. JCoast is available at the project homepage.

## Background

The sequencing of genomes and metagenomes has become a standard technology in molecular biology. Currently, over 700 sequenced genomes of bacterial and archaeal origin are publicly available [1]. Initiatives such as the Community Sequencing Program at the Joint Genome Institute (JGI), the Microbial Genome Sequencing Project funded by the Gordon and Betty Moore Foundation, or collaborations with Genoscope, enable researchers worldwide to get their genome or metagenome of interest easily sequenced. With the acceptance of a sequencing project, initial bioinformatic support is often granted through web-based systems, such as the Integrated Microbial Genomes (IMG and IMG/M) system [2] or Magnifying Genomes [3] to give two examples. To cope with the flood of data generated by community sequencing projects such as the Venter cruises [4,5], the CAMERA (Community Cyberinfrastructure for Advanced Marine Microbial Ecology Research and Analysis) consortium was recently established providing access to both the data and pre-computed information [6].

Standardised steps for data processing in (meta)genome analysis are highly appreciated for their ability to make results comparable and the processing transparent. Nevertheless, after the first round of data mining using web-based annotation systems, specific requests by the biologists typically arise, which ask for alternative views of the data. To deal with such demands, full access to the tools and databases is required. This is best handled through the use of "rich clients", which take full advantage of the native facilities of the user's computer. Making use of existing graphics hardware acceleration, rich clients can serve as graphical front-end to display complex and interactive visualisations [7]. One of the most popular, stable, flexible, and publicly available genome visualisation tools is Artemis [8]. Although, it can be extended for computations, it lacks a central storage system and is therefore, in most cases, only used as a viewer for genomic data.

A "state-of-the-art" infrastructure for (meta)genome analysis should be based on a relational database system that stores and organises assembled DNA sequence data, gene predictions, results from automatic analysis, and manual annotations [9]. The analysis should integrate similarity searches against a variety of different data sources based on established algorithms to get a comprehensive overview of the available information for each gene and gene family. Consistent data processing and storage is a prerequisite for flexible data analysis, which is essential when addressing specific requests of the biologists. Project and user management is also necessary to organise data access on all levels (administrator, annotator, guests). In 2003, the GenDB system [10] was released as an open source solution for high quality whole genome annotation. The system relies on a relational database system for back-end storage and takes advantage of Grid technology for massive distributed computing. In addition to project and user management, GenDB offers visualisation, annotation, and search capabilities via a web front-end. GenDB has already been successfully used in many annotation projects [e.g. [11-13]] and adopted by the Network of Excellence "Marine Genomics Europe" [14] as their standard tool for genome analysis.

Although equipped with an advanced backbone for data processing and storage, the available GenDB web-visualisation and analysis capabilities were not able to cover all user-specific requests such as parallel analysis of several genomic sources or a sortable tabular representation of the gene content. Further modules for the calculation of group-specific genes, COG statistics, advanced search functionalities, as well as gene grouping were continuously requested.

In order to address this issue, we developed JCoast, a Comparative Analysis and Search Tool for prokaryotic genomes. JCoast is a standalone tool that makes use of the standardised genome processing and storage system, GenDB. JCoast offers individual and cross genome- and metagenome analysis by handling several projects simultaneously. It provides a graphical user interface (GUI), and an application programming interface (API), including a plug-in facility for user extensions. JCoast can also work on local databases following the GenDB schema, independently of a full GenDB installation. It is publicly available and can be easily installed using the Java Webstart technology. The low system requirements, especially when pre-computed databases are used, the very limited need for maintenance, combined with highly flexible data analysis options, leaves the biologists to concentrate on biological questions rather than solving computation problems.

## Implementation

JCoast is written in the platform-independent, object-oriented programming language, Java [15]. It can be started using the Java Web Start technology, which automatically downloads and installs the software locally. This ensures the user to always get access to the latest version available. Alternatively, it can be downloaded and installed manually.

JCoast offers two entry points to access the genomic data and bioinformatic results:

i. the GUI, which is implemented with the Java-Swing extension, SwingX [16]. The GUI provides all functions necessary to analyse, search, and manipulate genomic information, and includes dedicated modules for addressing e.g. group specific genes (GSG) or comparative statistics based on profile-HMMs.

ii. the API, which provides object relational mapping to the underlying database and specific classes for advanced searches, data mining and statistics.

The JCoast source code is organised in:

i. the 'api' package, which contains all classes describing the core functionality;

ii. the 'gui' package, which contains all classes describing the graphical user interface and related inherited functionalities;

iii. the 'scripts' package, which contains ready to use methods for project maintenance, time consuming calculations (e.g. calculation of reciprocal best matches), and specific data transfer methods, such as the export of the database in NCBI Sequin format for genome data submissions.

By default, JCoast supports five bioinformatic tools: BLAST [17], Pfam [18], InterPro [19], SignalP [20], and TMHMM [21], and offers a direct access to the Geographic-BLAST tool provided by megx.net [22].

The current JCoast implementation relies on MySQL 5.0 [23] and a GenDB 2.2 compatible database schema. A detailed description of the GenDB system can be found in Meyer *et al.*, 2003 [10].

The following extensions to the GenDB database schema are necessary to support all features offered by JCoast:

### Gene groups

JCoast offers assigning a set of genes into "gene groups". This allows visualisation and analysis of the (meta)genomic information across projects. In order to store gene groups in JCoast, the GenDB database model needs to be extended by two tables, *Gene_Group *and *Gene_Group_Region*. *Gene_Group *contains the description of the group, *Gene_Groups_Region *contains the corresponding genes belonging to a defined *Gene_Group*.

### Codon offset

Partial protein coding genes that result from missing start codons are common in draft- and metagenomic datasets. To handle this, the GenDB database needs to be extended by the table *Region_CDS_Codon_Offset*. This ensures that the translation will always start on the first complete codon.

## Results

### The Graphical User Interface (GUI)

**Figure 1:**
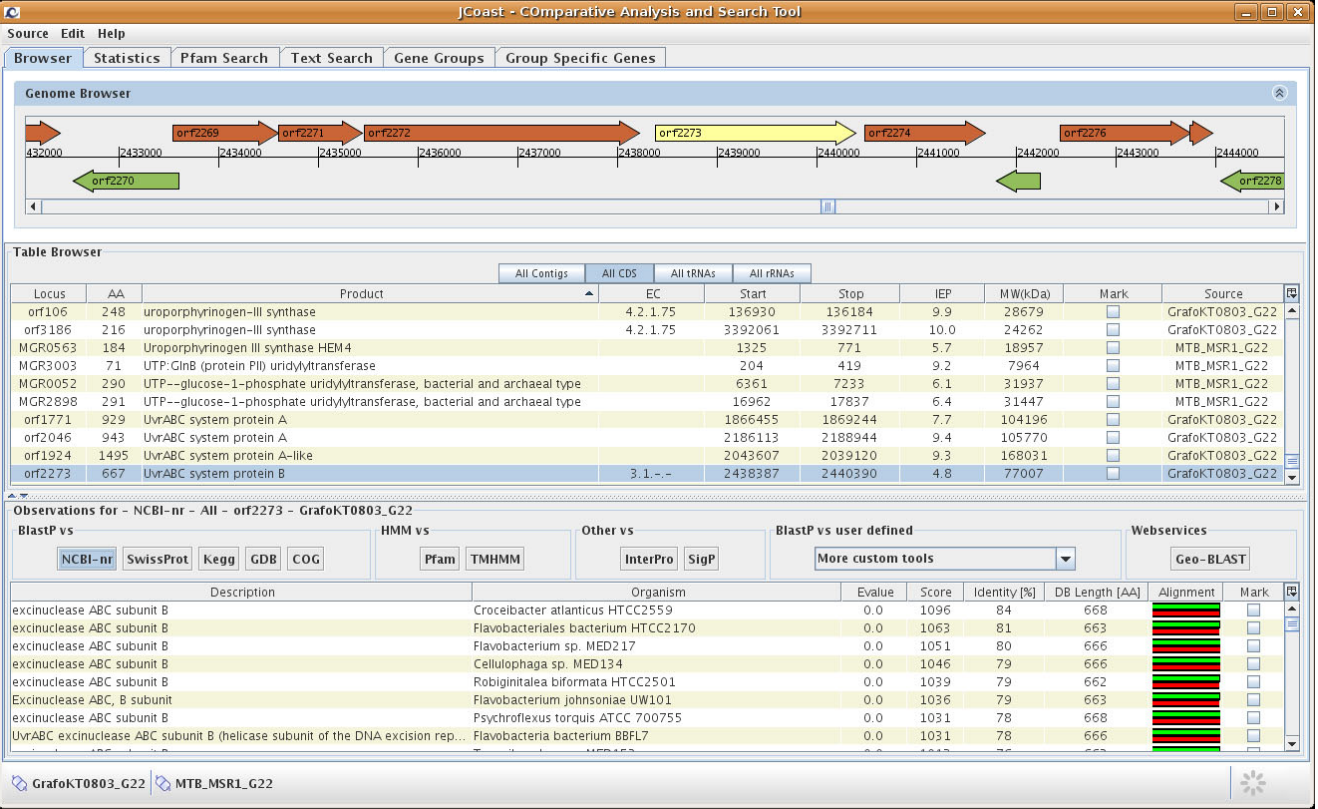
**JCoast overview**. The JCoast main window is separated into three panels, the 'Genome Browser' on top, the 'Table Browser' in the middle, and the 'Observation Browser' at the bottom. The 'Genome Browser' provides a graphical representation of genes on the genomic or metagenomic contigs under investigation. The 'Table Browser' displays different types of regions (CDS, contig, tRNA and rRNA) belonging to a project. A button panel implements rapid switching between regions. The 'Observation Browser' at the bottom displays the different similarity search results for a CDS.

The main window of JCoast is composed of six modules (Fig 1). The 'Browser' module is the central component of JCoast, which is re-used by most other modules to display results. It is separated into three panels, the 'Genome Browser' on top, the 'Table Browser' in the middle, and the 'Observation Browser' at the bottom.

The 'Genome Browser' provides a graphical representation of genes on the genomic or metagenomic contigs under investigation. It is directly linked to the 'Table Browser' where the corresponding region is shown. The 'Table Browser' displays different types of regions (coding sequence (CDS), contig, tRNA and rRNA) belonging to a project. Where a project is defined as all contigs belonging to e.g. a single organism or a metagenomic sample. A button panel implements rapid switching between regions. The panel also offers the ability to extract sequence information for a single gene, region, or for a whole project, as either amino acids or nucleotides. For each CDS entry, an 'Annotation dialog' can be entered. This dialog allows annotation of the gene products, the EC numbers, gene names, and additional comments for each entry. All annotations are immediately stored and subsequently available for other annotators. With the history function of GenDB all user changes are tracked. Additionally, regions can be deleted via the panel. The 'Observation Browser' at the bottom displays the different similarity search results for a CDS. Depending on the selected tools, additional functions are available for each entry. For example, every similarity search result is referenced to its original entry in public repositories (e.g. GenBank), if available. The public entry is shown in the standard browser of the host system.

**Figure 2:**
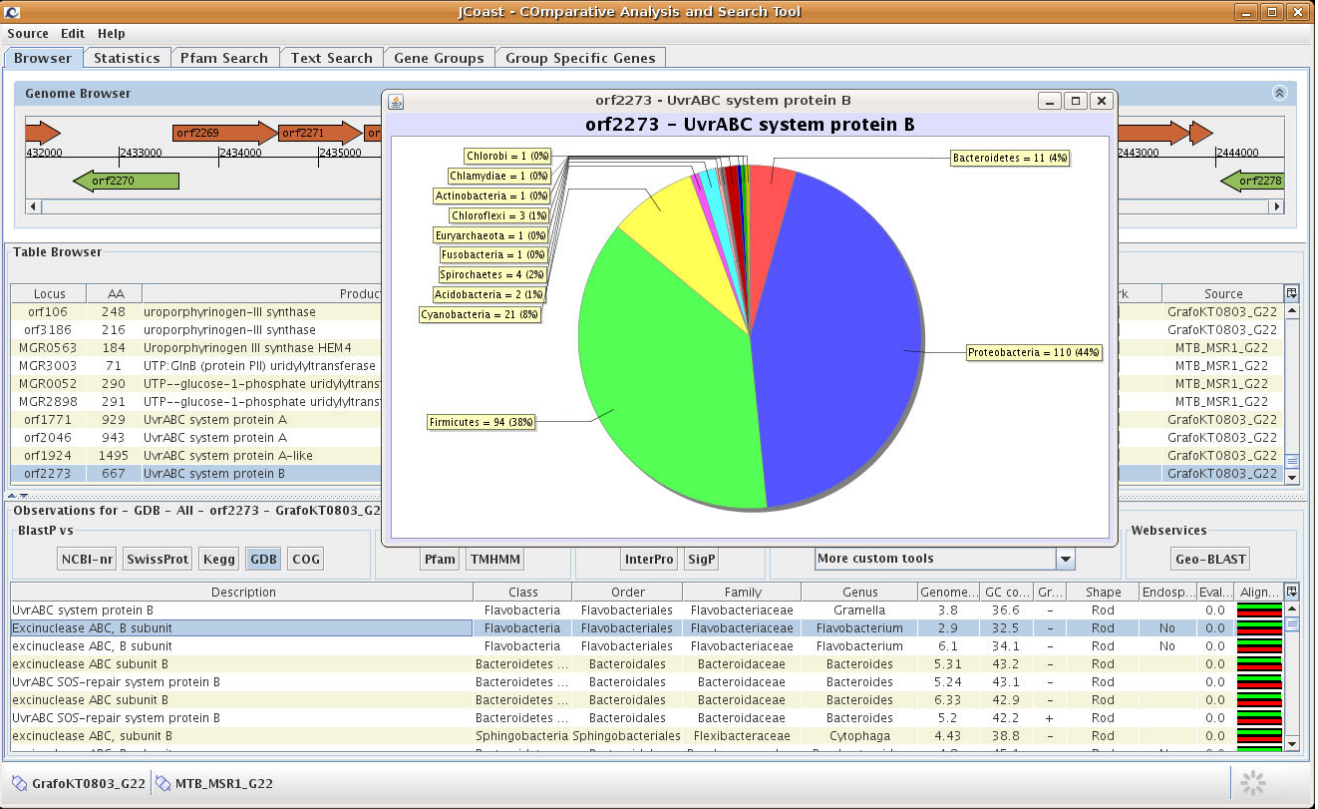
**Taxonomy distribution chart**. JCoast uses the database genomesDB for the calculation of GSGs and for drawing taxonomy distribution charts. For each CDS such a chart can be calculated on the fly, based on different taxonomic levels e.g. phylum, class, order, family or species. In addition also contextual information can be used for this calculation.

For the similarity search results against genomesDB (see below for more details), preconfigured charts are available for visualisation. For example, the taxonomic distribution analysis of BLAST hits can be shown in a pie chart, ordered either by phylum, family, order, class, or species (Fig 2).

All tables have a common set of functions to enhance the usability:

i. an alphanumeric sorter for each column of the table.

ii. the column control button in the upper left corner of each table; this button enables the user to hide and unhide a column within the table.

iii. a panel for text search in order to search within the visible content of the tables.

The module 'Statistics' includes three kinds of "on-the-fly" calculations based on:

i. Cluster of Orthologous Groups of Genes (COG) [24], which counts the absolute number of genes belonging to one of the COG categories.

ii. Pfam [18], which counts the absolute occurrence of a defined Pfam model in a project.

iii. project content, which calculates statistics about the number of CDS, contigs, tRNAs, rRNAs, nucleotide usage and coding percentage.

The 'Text Search' module has been designed for string and regular expression searches in annotations, comments, gene names, EC numbers (Annotation Search). Within the complex set of similarity search results (Observation Search); the search is filtered by applying an E-value cutoff. The results view makes use of the main browser panel and displays matching regions as subsets.

**Figure 3:**
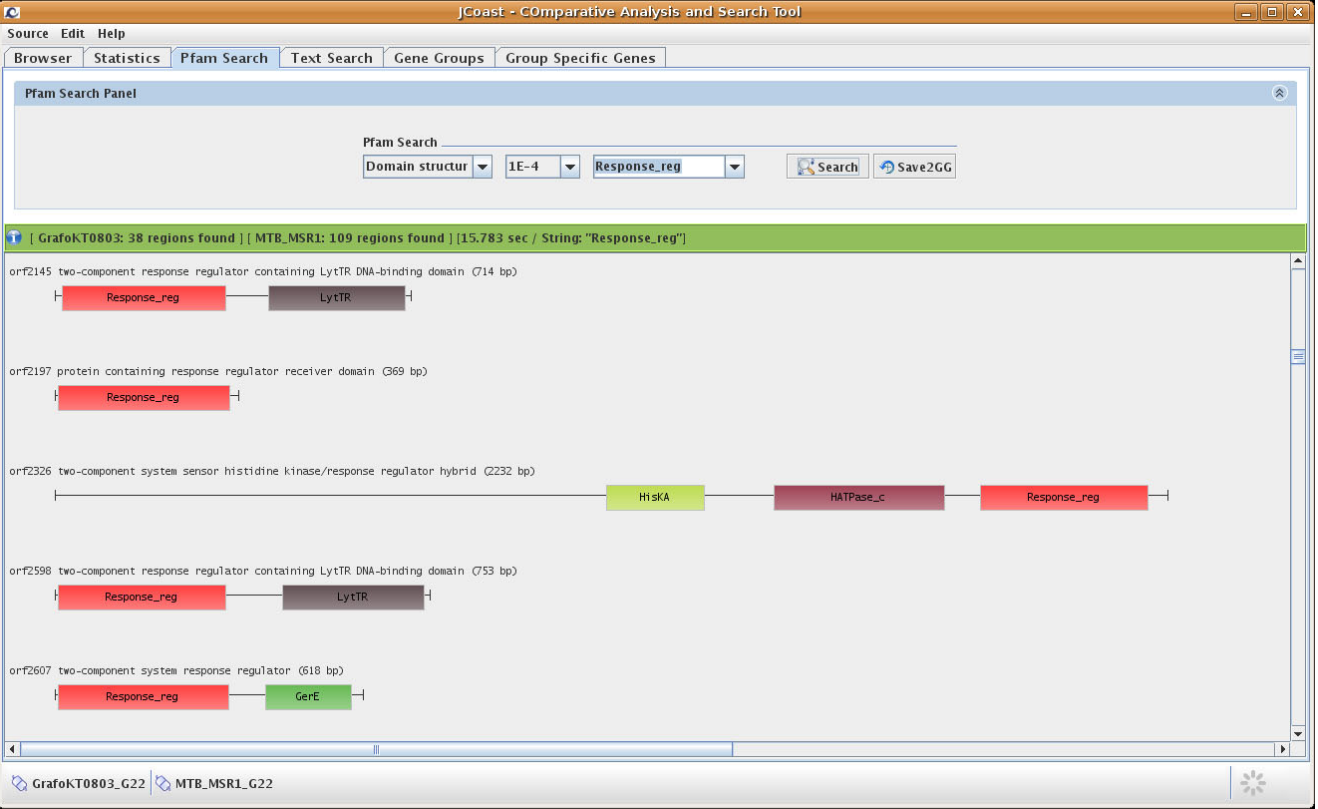
**Pfam model search**. JCoast supports extensive Pfam model search functionality, including graphical domain structure display.

The 'Pfam Search' module allows searching within Pfam models by applying an E-value cutoff. This allows, for instance, consistent cross genome comparisons. The results can be displayed as a subset of regions or as a graphical output of all Pfam models of each CDS (Fig 3).

The 'Group-Specific Genes' (GSG) module was designed to enable the researcher to search for genes with a limited occurrence in a given taxonomic group or any group defined by the user. This module is based on the custom database, genomesDB (see below). Each CDS of a reference genome is tested for group-specificity by looking at the observations produced by similarity searches on the protein level against the custom database, genomesDB. By definition, a GSG shows significant similarity only to genes in the same taxonomic unit. Therefore, an E-value cutoff was implemented to evaluate significance. To be group-specific, a CDS must have at least one "in-taxon" observation and no "out-of-taxon" observations below the defined threshold. Self-hits are filtered out and can be adjust within the preferences.

### The 'genomesDB' database

GenomesDB is a custom designed relational database, which includes a Java interface for maintenance. It is build from the proteome FASTA files obtained by the NCBI Reference Sequences database (RefSeq) for all fully sequenced bacterial and archaeal genomes (621 genomes, Jan 2008). Each genome, chromosome, and protein in the database is tagged with a unique internal numerical identifier. In addition, taxonomic and contextual information are parsed from NCBI Entrez Genome Project database. For every entry, taxonomic information is collected for the corresponding kingdom, phylum, class, order, family, genus and species. Further contextual data available pertain to genome size, guanine-cytosine content, Gram staining, shape, arrangement, endospores formation, motility, salinity, oxygen, habitat and temperature range. Genomes of interest can be selected for export via the interface, including the protein sequences as a multiple sequence FASTA file.

In contrast to the general-purpose database NCBI-nr, the focus of genomesDB is to provide manually curated phylogenetic affiliations, plus as much additional contextual information as possible. The database is used by JCoast to determine GSGs and to calculate distribution charts of selected properties. The current version of genomesDB can be downloaded from the JCoast homepage.

### Web-Service: Geographic-BLAST

To allow researchers to systematically study the geographic distribution of particular genes in the environment, a click on the 'Geographic BLAST' button in JCoast starts a remote BLAST search of the database for marine ecological genomix (megx.net) [22] for a selected gene. The results are shown on the Genomes Mapserver, which integrates sequence data with contextual information, such as physical, chemical, and biological data based on geography. In addition to the geographical distribution of particular genes, statistics are provided pertaining to the presence/absence and abundance of the gene of interest with respect to sampling sites and environmental conditions.

### The Application Programming Interface (API)

The JCoast core API comprises strictly defined objects of all the important GenDB database tables. All sequence related features, such as contigs or CDS definitions, annotations, and bioinformatic tool results are encoded within these Java objects. The core API also contains the complete SQL code, which is required to communicate with the database. The building of all required Java objects has been merged into a single class. This encapsulation renders JCoast flexible, making it possible to work on data sources other than GenDB. The JCoast core API is used extensively by the graphical user interface and the 'scripts' package, which is included in the JCoast source code. The classes within this package deliver an easy to use environment, mainly designed for users with little Java programming experience that want to use JCoast to address complex biological questions. The 'scripts' package includes template classes, which manage the database communication and user identification issues for the user. It also includes ready to use classes for maintaining projects, exporting data, and performing statistical calculations. Many of these classes are able to store the results directly in the database as 'GeneGroups' for subsequent evaluation of the results using the JCoast GUI.

### Case studies

JCoast has already been extensively used and evaluated in recently published comparative genomic and metagenomic projects.

In the field of genomics, JCoast was used to analyse the finished genome of the marine Bacteroidetes *Gramella forsetii *[25] in the context of 15 other Bacteroidetes draft sequences provided by the Moore foundation. Extensive analysis was successfully performed on the draft genome of *Magnetospirillum gryphiswaldense *strain MSR-1 for comparison with three other draft genomes of magnetotactic bacteria [26]. Aside from standard annotation support in both projects, detailed statistical analysis of the presence/absence and abundance of specific Pfam profiles in the genomes was performed to identify specialisation and niche adaptation of the organisms. Cross-comparisons of all genes within the phenotypic group of magnetotactic bacteria revealed a set of group specific genes that are now the subject of targeted lab experiments. The implementation of the group specific genes module, in combination with the genomesDB database, proved extremely helpful by significantly accelerating the transition from *in silico *predictions to lab work. Furthermore, the system was used to assist in the annotation and ongoing comparative analysis of *Congregibacter forsetii *KT 71 [27].

In the field of metagenomics, a prototype of the software was used to analyse and compare 30 Mb of DNA on 511 scaffolds (comprising 21,077 ORFs) the symbiotic community of the marine oligochaete, *Olavius *sp., sequenced by a community shot-gun approach [28], as well as 9 Mb of DNA on 7,860 scaffolds from single filaments of *Beggiatoa *sp. determined by combined genome amplification and pyro- and Sanger sequencing [29]. Both projects had a challenging and heterogeneous set of short to medium sized DNA scaffolds and contigs that needed to be analysed. The marginally low quality of the data was problematic, and led to the development of the codon offset table to cope with partial genes. Moreover, JCoast is currently used to analyse Fosmid-sized clone libraries from different marine sampling sites.

In all projects, the system was able to assist the biologists to generate results faster by providing custom-tailored solutions. On the other hand, close connection to the users provided valuable feedback for software enhancements.

## Discussion

Many data mining, annotation, and visualisation systems have been developed over the last years, each with their advantages and disadvantages, for a review see [30,7]. In addition, several stand-alone tools have been introduced, which rely on object-oriented programming languages (e.g. Strainer: Software for analysis of population variation in community genomic datasets [31] or MetaLook: a 3D visualisation software for marine ecological genomics [32]). They have been designed to facilitate the exploration and analysis of highly specific datasets. Moreover, they are user-friendly and offer simple installation procedures. A shift from process to object oriented programming languages is a current trend in biologist-centric software development.

The JCoast software tool offers the unique combination of a standardised, open source relational database system in the back-end and a user-oriented rich-client in a lightweight, stand-alone solution. The common usage of the GenDB system in academia and industry has made it easy to find a collaboration partner providing initial data processing and database access. The company Ribocon GmbH already offers custom tailored genome analysis in GenDB/JCoast format on a commercial basis [33].

The possibility to run JCoast locally with either on site or remote access to pre-computed databases frees the biologist from the need to acquire specialised knowledge about how to install and maintain complex annotation pipelines, while taking advantage of an advanced database structure and a tightly-linked graphical user interface. As a result, the biologists can focus on their research with no or minimal programming efforts.

With the advent of next generation sequencing technologies even independent working groups or individual researchers can get easy access to genomic sequence data. In such a case, researchers often go for specific genes of interest in a defined set of genomes or metagenomes, rather than to perform a time-consuming comprehensive annotation of an entire (meta)genome. Therefore, easy to use and flexible data mining software systems will most likely be favored over complex annotation systems. Biologist-centric software tools such as JCoast facilitate these tasks by providing components with sophisticated bioinformatic functionalities without prior programming knowledge of the biologist [34].

Furthermore, the handling of sensitive data, as it is often the case in commercial applications, demands for locally installed software systems. Nevertheless, there is no doubt that the analysis of thousands of genes and large scale comparisons with published data is not trivial and will always require an appropriate cyberinfrastructure.

Currently, JCoast is primarily used for the analysis of prokaryotic genome data. In general, the GenDB system supports the analysis of eukaryotic data as well, but handling the additional information for such projects is not implemented in JCoast at the moment. Several extensions are planned for the future, e.g. including an importer allowing the user to import standardised sequence description files which currently can only be done using the import capabilities of the GenDB backbone. A project and user management system will be necessary to enhance usability in this respect. Common genome linguistic methods (GC-skew, oligonucleotide statistics), data quality checks for 454/Solexa [35] sequences and further incorporation of contextual (meta)data standards are envisioned.

## Conclusion

JCoast is a biologist-oriented graphical software tool that provides a powerful API to manipulate and data mine genomic information and bioinformatic results using the Java object-oriented programming language. The GUI is able to handle large metagenomes, as well as minimally assembled single genome projects. It provides a full-featured genome browser and sophisticated statistical and search functionalities. JCoast is developed as an extension to the GenDB back-end, but can also be used as standalone software. Pre-computed databases can be gained through an academic collaboration or by a dedicated service company [33]. This lightweight software solution allows the biologist to concentrate on transforming genomic data into biological knowledge with minimal programming experience. JCoast has been successfully applied to several genome and metagenome projects and has proven to be both stable and easy to use. The JCoast software tool is publicly available from the project website via Java Webstart or as a Kubuntu [36] JCoast-LiveCD. The source code is available upon request from the authors.

## Availability and Requirements

- **Project name**: JCoast – Comparative Analysis and Search Tool

- **Project homepage**: 

- **Operating systems**: Linux and Windows

- **Programming language**: Java JRE 1.5 or higher

- **Other requirements**: Pre-computed GenDB V2.2 MySQL database

- **License**: GNU General Public License version 3 (GPL3)

## Authors' contributions

MR designed and implemented most of the API and GUI. TL implemented the core search functionalities of the API and GUI. IK implemented the extraction of group-specific genes and helped with genomesDB. RK gave expert advises for programming design and code optimisation. MBD contributed necessary classes for the calculation of statistics of large data sets and helped with the website. JP tested the GUI and helped to improve it. FOG supervised the work and helped with writing the manuscript.
